# Protective Passive Immunity in *Escherichia coli* ETEC-Challenged Neonatal Mice Conferred by Orally Immunized Dams with Nanoparticles Containing Homologous Outer Membrane Vesicles

**DOI:** 10.3390/vaccines8020286

**Published:** 2020-06-08

**Authors:** Jose Matías, Yadira Pastor, Juan M. Irache, Carlos Gamazo

**Affiliations:** 1 Department of Microbiology and Parasitology, Institute of Tropical Health, University of Navarra, 31008 Pamplona, Spain; jmatias@alumni.unav.es (J.M.); ypastor@alumni.unav.es (Y.P.); 2 Department of Pharmaceutical Technology and Chemistry, University of Navarra, 31008 Pamplona, Spain; jmirache@unav.es

**Keywords:** vaccine, outer membrane vesicles, ETEC, *Escherichia coli*, nanoparticles

## Abstract

Enterotoxigenic *Escherichia coli* (ETEC) strains are a major cause of illness and death in mammals, including neonatal, recently weaned pigs and infant human beings. We have previously shown that outer membrane vesicles (OMV) obtained from ETEC serotypes encapsulated into zein nanoparticles, coated with a Gantrez-mannosamine polymer conjugate (OMV-NP), were immunogenic in mice and sows. In the present study, we show that pups from vaccinated mice were protected against ETEC F4 serotype challenge through maternal passive immunization. OMV from F4 cultures were collected and characterized. Two-week-pregnant BALB/c mice were orally immunized with a single dose of vesicles (0.2 mg) either free (OMV) or encapsulated into nanoparticles (OMV-NP). Evaluation of the antibodies in serum (IgG1, Ig2a or IgA) and feces (IgA) of dams immunized with OMV-NP revealed an enhancement of specific immunogenicity. The antibody response conferred by the nanoparticle adjuvant was also correlated with IL-6 and IL-10 splenic levels. Each mother was allowed to feed her progeny for one week. Suckling pups presented specific IgA in feces demonstrating their passive immunization through colostrum intake. Two weeks after the pups were born, they were infected orally with a single dose of F4 *E. coli* (1.2 × 10^8^ CFU/pup). Results showed that 70% of the pups from dams immunized with OMV-NP were protected. In contrast, 80% of the pups from dams immunized with free OMV died as a result of the experimental challenge. These findings support the use of zein nanoparticles coated with a Gantrez-mannosamine shield as adjuvant delivery system for the oral immunization during pregnancy to confer immunity to the offspring through maternal immunization

## 1. Introduction

Enterotoxigenic *Escherichia coli* (ETEC) strains are relevant pathogens of both humans and farm animals [1,2]. In particular, ETEC associated diarrhea causes a major percentage of the children annual death rate (525/100,000 children) but, however, there is no licensed vaccine against ETEC for humans [3]. Newborn and weaned animals are extremely susceptible to ETEC infections due to their genetic immunodeficiency at birth, and antimicrobial immunity depends on the mother Maternal immunity provides protection mainly through the transference of antibodies via placenta and through colostrum and milk. However, in some animal species there is not an efficient maternofetal transfer of immunoglobulins via placenta and receive passive immunity predominantly postnatally through lactation [4,5]. This maternally derived immunity must provide sufficient protection long enough while the infant immune system gradually matures and develops its own active immunity. Maternal immunization during pregnancy is one of the recommended strategies to improve infectious diseases in infants. To achieve this objective, the vaccine formulations must be able to induce a strong mucosal immune response [6]. Among the different mucosal routes, the oral vaccination is preferred due to its safety and easy way of administration. However, it must face several challenges. Oral immunization requires the successful delivery of the intact and active antigen to the intestine avoiding degradation through the harsh environment in the stomach. Polymeric nanoparticulate delivery systems (NP) are well recognized adjuvants that can reach those goals [7,8,9].

The adequate selection of the polymer deeply determines the adjuvant effect. In this context, nanoparticles based on the copolymer of methyl vinyl ether and maleic anhydride (PVM/MA) have demonstrated their efficacy as adjuvants to induce Th1 immune responses. In fact, these poly(anhydride) nanoparticles induce innate immune responses mediated by a TLR-2 and TLR-4 dependent manner [10,11].

We have previously shown that outer membrane vesicles (OMV) obtained from ETEC serotypes encapsulated into zein nanoparticles coated with a Gantrez-mannosamine polymer conjugate (OMV-GM-NPZ) were immunogenic in mice and sows. In the current study, we test the efficacy of one single oral dose of *E. coli* OMV encapsulated into NP nanoparticles administered in pregnant mice to confer protective immunity to the suckling offspring.

## 2. Materials and Methods

### 2.1. Chemicals

Poly (methyl vinyl ether-co-maleic anhydride) or poly (anhydride) (Gantrez**^®^** AN119) was supplied by Ashland (Ashland, OR, USA). Mannosamine hydrochloride, zein, mannitol, lysine, tween 20, 2-bromoethylamine-hydrobromide, trifluoroacetic acid and bovine serum albumin (BSA) were purchased from Sigma-Aldrich (Madrid, Spain). Sucrose was supplied by Fagron (Barcelona, Spain). Ethanol formaldehyde, sodium hydroxide and dimethyl sulfoxide (DMSO) were supplied by Panreac (Barcelona, Spain). Acetone was obtained from VWR-Prolabo was provided by Invitrogen (Carlsbad, CA, USA). Tryptic soy broth (TSB) was obtained from bioMérieux (Marcv L’Etoile, France). RPMI 1640 and fetal bovine serum were obtained from Gibco-BRL (Thistle Scientific, Glasgow, UK). Coomassie brilliant blue and sample buffer were purchased from Bio Rad (Madrid, Spain).

### 2.2. Preparation and Characterization of the OMV Vaccine Complex from Escherichia Coli

The vaccine complex consisted of OMV isolated from the ETEC F4 serotype (CECT 71709, Valencia, Spain). Vesicles were purified from a method adapted from Camacho et al. [12]. Bacteria were incubated in TSB under shaking to early stationary phase (37 °C, 125 rpm) and then were inactivated with a solution of binary ethylenimine and formaldehyde (6 mM BEI-0.06% FA, 6 h, 37 °C). Cells were discarded by centrifugation (10,000× *g*, 20 min) and supernatant filtered through a 0.45 µm Durapore PVDF filter (Merck KGaA, Darmstadt, Germany) and purified by tangential filtration using a 300-kDa concentration unit (Merck KGaA, Darmstadt, Germany). The retenant was finally lyophilized.

Total protein content was determined by the method of Lowry, with BSA as the standard. Lipopolysaccharide (LPS) content was determined by KDO assay. The protein composition was determined by proteomic analysis, using a mass spectrometry from three independent batches. Briefly, one milligrams of sample was subjected to trypsin digestion. Peptides were solubilized in 1% trifluoroacetic acid and further extracted using a C18 column (Pierce C18 Spint Tips, ThermoFisher, Waltham, MA, USA) following the manufacturer protocol. Extracted peptides were dried in a speed-vac and subjected to a mass spectrometry analysis. MS/MS data acquisition was performed using Analyst 1.5.2 (Sciex, Madrid, Spain) and spectra files were processed through Protein PilotTM Software (v 5.0 Sciex, Madrid, Spain) using ParagonTM Algorithm (Sciex, Madrid, Spain) for database search, ProGroupTM (Sciex, Madrid, Spain) for data grouping, and searched against the UniProtKB proteome reference. False discovery rate was performed using a non-lineal fitting method and a result group file was created reporting only results with 1% Global false discovery rate or better. The peptide quantification was performed using the Progenesis LC–MS software (Nonlinear Dynamics, Newcastle upon Tyne, UK). Runs were automatically aligned, and they were manually supervised. The peptide identifications were exported from Protein Pilot and imported in Progenesis LC–MS where they were matched to the respective features. For quantification, only unique peptides were included, and the total cumulative abundance was calculated by summing the individual abundance of all peptides assigned to each protein.

### 2.3. In Vitro Cytotoxicity Studies in Macrophages

The MTT colorimetric assay was used to test the macrophage cytotoxicity induced by OMV. The MTT assay depends on the mitochondrial reductive capacity of living cells to reduce 3-[4**–**dimethylthiazol-2-yl]-2, 5 diphenyltetrazolium bromide (MTT) into formazan (Promega, San Luis Obispo, CA, USA). The macrophage RAW 264.7 cell line was used for in vitro cytotoxicity analysis. A total of 10^5^ RAW macrophages were seeded into in a 96 well plate (24 h, 37 °C, 5% CO_2_, 95% humidified air). After washing with phosphate-buffered saline (PBS). OMV suspended in cell medium were added to the wells to reach a final concentration of 0.75, 3, 6 or 12 µg/mL. As a positive control, cytotoxic Triton × 100 in cell medium (0.5 % v/v) was used. Cells were incubated for 24 h. After incubation, medium was removed and 100 mL of MTT solution was added (3 h, 37 °C, 5% CO_2_, 95% humidified air). Then, 200 mL DMSO was added to dissolve the reduced formazan product generated by live cells. Then, the plate was shaken for 10 min at room temperature, and absorbance was measured at 540 nm using a microplate reader (Agilent, Santa Clara, CA, USA). Cell cytotoxicity was assessed as a percentage in relation to controls.

### 2.4. In Vitro Evaluation of OMV in Macrophages and Splenic Cells

The production of nitric oxide (NO) is characteristic of the classical macrophage activation, M1 phenotype. In order to characterize the ability of OMV to modulate the macrophage maturation to M1, OMVs were incubated with the macrophage RAW 264.7 cell line. In brief, 10^5^ RAW cells per well were seeded in 96-well polystyrene plates (Costar**^®^** Merck KGaA, Darmstadt, Germany) and allowed to adhere for 24 h at 37 °C in a humidified atmosphere containing 5% CO_2_. Afterwards, three different concentrations (10 µg/mL, 40 µg/mL or 160 µg/mL) of OMV-F4 or OMV-F18 were added in triplicates, and cells were incubated for 24 h. Then, 50 μL of culture supernatants were incubated with an equal volume of Griess reagent (Panreac, Barcelona, Spain; 0.5% sulfanilamide and 0.05% naphthylene-diamide dihydrochloride in 2.5% H_3_PO_4_) for 30 min at room temperature. Absorbance was measured using a Multiskan EX (ThermoFisher, Waltham, MA, USA) microplate photometer plate reader (Thermo Fisher Scientific Slu., Madrid, Spain) at 540 nm. The nitrite concentration in the medium was determined from the calibration curve obtained by using different concentrations of sodium nitrite.

In order to determine the OMV stimulation capacity to drive a Th2 (IL-4 secreting subset) or Th1 (IFNγ secreting subset) response, spleen cells from naïve mice were in vitro stimulated with OMV. Four naïve BALB/c were sacrificed by cervical dislocation, and spleens were removed and placed in RPMI 1640 media under sterile conditions. Spleens were homogenized and centrifuged (450× *g*, 10 min), the supernatants were discarded, and pellets washed twice in 10 mM PBS (pH 7.4). The cellular pellets were resuspended in RPMI 1640 medium (Gibco-BRL, Cheshire, UK) supplemented with 10% of fetal bovine serum (Gibco-BRL, Cheshire, UK) and 500 μL of penicillin (10,000 units/mL)-streptomycin (10,000 µg/mL) solution (Gibco-BRL, Cheshire, UK). In a 96-well microtiter plate (Costar**^®^** Merck KGaA, Darmstadt, Germany), cell suspensions were added at 10^5^ cells/well, along with 40 μg/mL of OMV using LPS from *E. coli* at 10 μg/mL as a positive control, and PBS as negative control. All wells were set up in triplicates. Cells were incubated (37 °C, 48 h, 5% CO_2_), supernatants were collected and the levels of IFN-γ and IL-4 released determined with a commercial sandwich ELISA kit (Biosource, Camarillo, CA, USA).

### 2.5. Preparation of OMV-Loaded Zein Nanoparticles

Nanoparticles were prepared in a two-steps process in which OMV were first encapsulated in zein nanoparticles and subsequently coated with a polymer conjugate obtained by the covalent binding of mannosamine to the copolymer of methyl vinyl ether and maleic anhydride (Gantrez**^®^** AN). In a first step the polymer conjugate between Gantrez**^®^** AN and mannosamine was synthetized. For this purpose, 1 g Gantrez**^®^** AN was dissolved in 120 mL acetone. Then, 50 mg mannosamine were added and the mixture was heated at 50 °C (400 rpm magnetic agitation, 3 h). The mixture was filtered, and the organic solvent eliminated under reduced pressure (BÜCHI Labortechnik AG, Flawil, Switzerland). The GM conjugate was characterized and the mannosamine content was calculated to be 21 µg/mg polymer.

In a second step, zein nanoparticles were obtained by dispersing 400 mg zein, 15 mg OMV and 66.7 mg L-lysine in 40 mL ethanol 70%. Nanoparticles were produced by the addition of 40 mL water in magnetic agitation. Then, 1 mL of a solution of GM in water (10 mg/mL) was added to the suspension of OMV-loaded nanoparticles and incubated for 30 min. Finally, 13.33 mL of and aqueous solution of mannitol (200 mg mannitol/100 mg zein) was added to the suspension of nanoparticles and the mixture was dried in a Spray-drier (BÜCHI Labortechnik AG, Flawil, Switzerland) under the following experimental conditions: inlet temperature of 90 °C, outlet temperature of 60 °C, spray-flow of 600 L/h and aspirator at the maximum capacity. The resulting nanoparticles were identified as OMV-NP, for OMV-loaded nanoparticles). Empty nanoparticles were prepared in the same way as described above but in the absence of OMV.

### 2.6. Characterization of the Nanoparticle Formulation

The size and zeta potential of nanoparticles were determined by photon correlation spectroscopy (PCS) and electrophoretic laser Doppler anemometry, respectively, using a Zetamaster analyzer system (Malvern**^®^** Instruments, Malvern, UK). Samples of nanoparticles were dispersed in ultrapure water at 25 °C in order to measure the diameter with a scattering angle of 90°.

To measure the amount of OMV encapsulated in nanoparticles, the dried nanoparticles were dispersed in water and centrifuged (10,000× *g*, 5 min). The supernatant containing mannitol and free OMV was removed and the pellet containing the nanoparticles resuspended in 70% ethanol. Another centrifugation resulted in pellet containing OMV and protein residues (10,000× *g*, 5 min). This pellet was resuspended in a mixture of 20% DMSO in 85% ethanol and the last centrifugation was performed. After removing the supernatant, we obtained the final pellet, which was used for further experiments.

To study the efficiency of encapsulation, SDS-PAGE and immunoblotting were performed. In brief, 10 mg/mL of loaded nanoparticles were suspended in ultrapure water and centrifuge (10,000× *g*, 20 min). Then, the supernatant was discarded, and the pellet resuspended in 10 mL of ethanol 70% and centrifuge 10,000× *g* during 20 min, and the sediment reconstituted in 10 mL of 20% DMSO–85% ethanol. After centrifugation under the same conditions, the pellet was resuspended in 100 µL of sample buffer (Tris-HCl 62.5 mM, pH 6.8; 10% glycerol; 2% sodium dodecyl sulfate, SDS; 5% β-mercaptoethanol and bromophenol blue) and boiled for 5 min. SDS-PAGE was performed in 12% acrylamide gels (Criterion XT, Bio Rad Laboratories, Madrid, Spain). The gels where stained with Coomassie brilliant blue (Bio Rad Laboratories, Madrid, Spain). The apparent molecular masses of the proteins of the samples were determined by comparison with standard molecular weight markers (Amersham Pharmacia Biotech, Little Chalfont, UK).

For the antigenicity studies, an immunoblotting assay was performed. The components separated electrophoretically were transferred from the gel to a nitrocellulose membrane (Whatman Protran**^®^**; Merck KGaA, Darmstadt, Germany, pore size 0.45 µm) using a semidry blotting system at 0.8 mA/cm^2^ for 30 min (Trans-Blot**^®^** SD Transfer Cell, BIO-RAD, Hercules, CA, USA). After the blocking of protein-binding sites with a blocking solution (PBS with 5% skimmed milk, overnight, room temperature), the membranes were washed 4 times with PBS-T and then incubated (4 h at 4 °C) with sera from pigs infected with F4 ETEC and diluted 1:500. The membranes were washed 4 times with PBS-T and then peroxidase (PO)-conjugated secondary antibody (GAP/IgG (Fc), HRP conjugate, 1:1000) was added for 60 min at room temperature. Finally, membranes were extensively washed with PBS-T and the antibody-antigen complexes were visualized after addition of a substrate/chromogen solution (H_2_O_2_/chloro α-naphthol).

### 2.7. Immunization Studies in Pregnant Mice and Challenge of the Offspring

Mice were treated in accordance with institutional guidelines for the treatment animals (Ethical Committee for the Animal Experimentation of the University of Navarra, ref. 138/16). Two-week-pregnant BALB/c mice (25 ± 1 g) were randomized in 4 groups of 3 animals. The mice were immunized orally with a single dose of OMV (0.2 mg) either free or encapsulated into nanoparticles (OMV-NP) or intraperitoneally with the commercial vaccines (Suiseng**^®^**, Lab. Hipra, Gerona, Spain). PBS was used as control. Pups were born one week after the immunization of pregnant mice. One week after birth, two weeks after mother’s immunization, the pups were weighted and infected orally with a single dose of F4 *E. coli* ETEC (1.2 × 10^8^ CFU/mouse) resuspended in 100 µL PBS. The mice were monitored along one week after the infection controlling the number of deaths and the symptoms. Finally, the survival pups were weighted and sacrifice by cervical dislocation.

#### 2.7.1. Specific Antibody Response

Blood and fecal samples from pregnant mothers were collected before immunization (day 0), the day of birth (day 6), the day of the infection (day 15) and the sacrifice day (day 22). In the case of pups, fecal samples were collected at days 7, 15 and 22 after dam’s immunization. Specific antibodies were determined by indirect ELISA. Briefly, microplate wells (Immuno-Maxisorp, Nunc**^®^**, Roskilde, Denmark) were coated with 10 µg/mL OMV in sodium carbonate-bicarbonate buffer (0.05 M, pH 9.6), and incubated overnight at 4 °C. The plates were blocked with PBS containing 3% BSA for 1 h at 37 °C. Fecal sample were treated with a protease inhibitor cocktail (Invitrogen, Thermo Fisher Scientific Slu., Madrid, Spain) and kept in PBS-3% milk at −20 °C until use. Serum or fecal samples from mice were diluted 1:100 or 1:10, respectively, in PBS in triplicate (1 h, 37 °C). After five washes with PBS Tween 20 buffer (PBS-T), the alkaline phosphatase-conjugated detection antibody, class-specific goat anti mouse IgG1/IgG2a/IgA (Merck KGaA, Darmstadt, Germany), was added for 1 h at 37 °C. The detection reaction was carried out by incubating the sample with H_2_O_2_-ABTS substrate-chromogen solution for 20 min at 37 °C. Absorbance was measured with an ELISA reader (Sunrise remote; Tecan-Austria, Groeding, Austria) at a wavelength of 405 nm.

#### 2.7.2. Cellular Immune Response

To determine the sort of cellular immune response elicited after immunization IL-6, TNFα and IL-10 were determined from splenic cells. Naive and immunized mice were sacrificed by cervical dislocation at day 21 after immunization and their spleens were removed and placed in RPMI 1640 media (GibcoBRL, Cheshire, UK) under sterile conditions. The spleens were grinded, and cells were pooled in one flask. The cellular suspension was centrifuged at 380× *g* for 10 min, the supernatant discarded, and the pellet washed twice with PBS. The splenocytes were resuspended in lysis buffer (NH_4_Cl 0.15 M, KHCO_3_ 10 mM and EDTA 0.1 mM) to eliminate erythrocytes, and after 2 min dissolved in RPMI 1640 to stop the reaction. This suspension was centrifuged (380× *g*, 5 min) and the pellet was resuspended in RPMI 1640 medium supplemented with 1 IU/mL penicillin, 1 µg/mL streptomycin and 10% fetal bovine serum (Gibco-BRL, Cheshire, UK). The lymphocyte suspension was added to 96-well round bottom microtiter plates (Iwaki, Shrewsbury, UK; 4 × 10^5^ cells/well) and received one of the following stimuli: F4-OMV (10 µg/mL) in a final volume of 200 µL/well. Negative control (PBS) and positive control (100 ng/mL + 4 µg/mL of PMA/Ionomicine used as mitogen) controls were used. The culture supernatants were collected for cytokine assay at 72 h after the stimulation and were kept frozen at −80 °C. Cytokines were quantified by using the kit BD Cytometric Bead Array Th1/Th2/Th17 CBA (BD Biosciences, San Jose, CA, USA) with a flow cytometer (Acoustic Focusing Cytometer Attune**^®^** Thermo Fisher Scientific Slu., Madrid, Spain)

### 2.8. Statistical Analysis

Analysis of variance (ANOVA) was employed to analyze data and when significant differences were found, the Tukey post hoc test was used to assess the difference between groups. *p*-values <0.05 were considered statistically significant.

## 3. Results

### 3.1. OMV Characterization

Isolated OMV obtained from ETEC strain in terms of morphology such as the range size were confirmed with previous reports [13] showing to be spherical (15–200 nm) without cell debris. The yield obtained in the process of isolating OMV was 23.0 ± 0.1 µg/mg, referred to the original cell culture wet weight. Protein content was 41.9% ± 2.6%, whereas the LPS content measured by KDO assay was 15.8% ± 1.9%.

**Figure 1:**
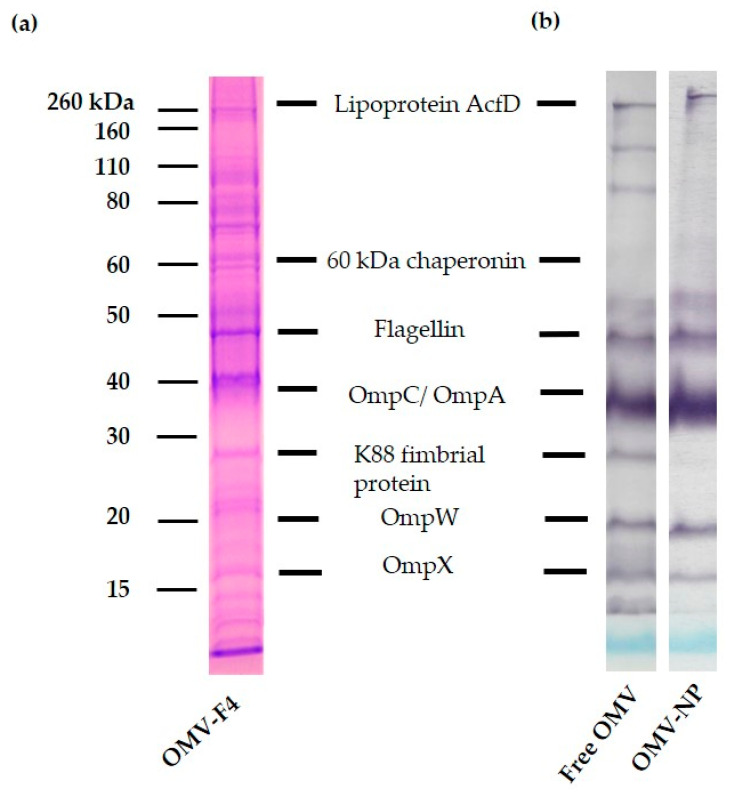
Protein profile and antigenicity of the outer membrane vesicle (OMV) encapsulated into nanoparticles. SDS-PAGE with Coomassie blue staining of OMV from F4 *Escherichia coli* (**a**). Molecular weight markers are indicated on the left in kDa. Immunoblotting using sera from infected piglets with F4 *E. coli*, showing the main antigenic proteins from OMV (**b**).

An SDS-PAGE was performed (Figure 1), and the identification of each band was confirmed by proteomic analysis. The results revealed that the OMV contained main immunodominant antigenic proteins of *E. coli* such as flagellin, OmpC/Omp and OmpW, among others (Figure 1). A complete quantitative proteome analysis was performed with the whole vesicles revealing that of the 664 OMV proteins, 31 were associated with pathogenesis and virulence, including proteins associated with attachment to host cells (e.g., flagellin, predicted protein and F4 fimbrial adhesins). Most of the proteins (52%) belonged to the outer membrane (e.g., OmpC, OmpA and OmpW) but also OMV contained components of the inner membrane (e.g., assembly factor BamA and porin protein NmpC) and cytosolic fractions.

### 3.2. In Vitro Toxicity and Evaluation of OMV in Macrophages and Splenic Cells

**Figure 2:**
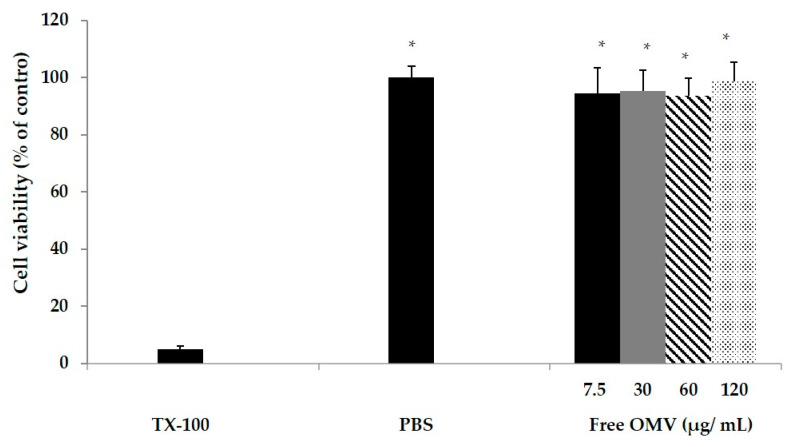
Effect of OMV on the mitochondrial activity (MTT assay) of RAW 264.7 macrophage cells. Figures indicate the percentage (%) of mitochondrial activity of murine macrophages RAW 264.7 after treatment for 24 h with different concentrations of OMV (μg/mL). The results are shown as the mean for triplicate assays ± SD (* *p* < 0.05).

MTT assays were performed to evaluate the toxicity of OMV in the RAW macrophage cell line. Results showed no toxicity at any of the tested concentrations of OMV (7.5 to 120 µg/mL; Figure 2). Positive control (Triton X-100 treated cells) and negative control (PBS treated cells) values were 5.9% ± 2.5% and 100%, respectively.

**Figure 3:**
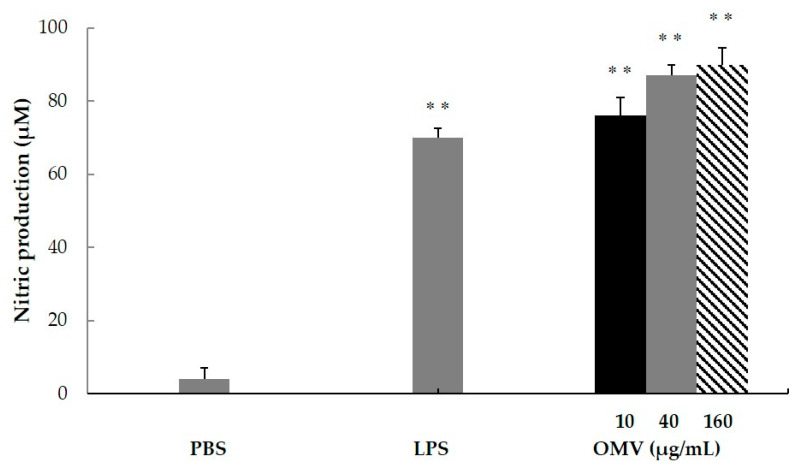
Macrophages generate nitric oxide in response to OMV-F4 *Escherichia coli* enterotoxigenic *Escherichia coli* (ETEC). The capacity of the OMV antigenic complexes to induce production of nitric oxide in the murine macrophage cell line RAW 264.7 was studied at three different concentrations (10, 40 and 160 µg/mL). The results are shown as the mean NO_2_^−^ levels for triplicate cultures ± SD. NO_2_^−^ levels were significantly greater (** *p* < 0.01) than those in supernatants from splenic cells incubated with culture medium alone.

The capacity of the OMV antigenic complexes to induce production of NO in macrophages was studied at three different concentrations (10, 40 and 160 µg/mL). Results demonstrated a significant NO production for OMV-F4 *E. coli* (Figure 3).

**Figure 4:**
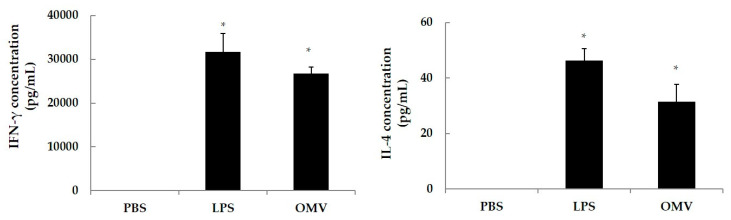
Cytokine production by spleen cells from non-immunized BALB/c mice stimulated in vitro with OMV. Spleen cells from naïve mice were in vitro stimulated with OMV (40 μg/mL), using LPS from *E. coli* at 10 μg/mL as a positive control and PBS as negative control. The levels of IFN-γ and IL-4 released were determined after 48 h treatment. The results are shown as the mean levels for triplicate cultures ± SD. Cytokine levels were significantly greater (* *p* < 0.05) than those in supernatants from splenic cells incubated with PBS.

In order to determine the degree of differentiation of splenic cells from naive mice after incubation with OMV, IFN-γ and IL-4 were determined in cell supernatants after exposure to OMV. Results demonstrated there were no significant differences between both OMV products, with high levels for IFNγ (Figure 4).

### 3.3. Characterization of Nanoparticles

The resulting OMV containing nanoparticles displayed a mean size of 132 ± 5 nm, with a polydispersity index of 0.104 and a negative zeta potential of –22 ± 1.7 mV. This last value was less negative than that of empty nanoparticles (–27 ± 0.8 mV; particle size, 138 ± 5.1 nm), suggesting the presence of antigenic components on the nanoparticles surface. Moreover, the payload of the resulting nanoparticles was calculated to be 9.2 µg OMV per milligram of nanoparticles. Immunoblotting studies, performed using sera from pigs infected with F4 ETEC, indicated that the OMV incorporation in nanoparticles did not alter their antigenic properties (Figure 1).

### 3.4. Immune Response in Vaccinated Mice During Pregnancy

**Figure 5:**
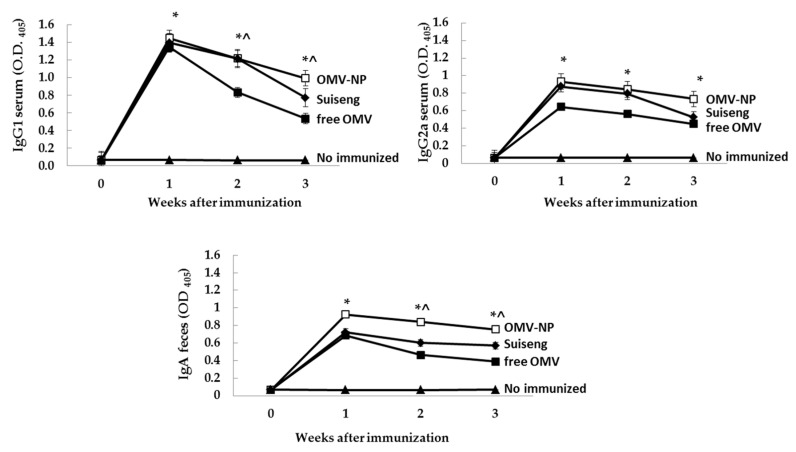
Antibody immune response induced after immunization of pregnant BALB/c mice. Two-week-pregnant BALB/c mice were immunized orally with a single dose of OMV (0.2 mg) either free or encapsulated into nanoparticles (OMV-NP) or intraperitoneally with the commercial vaccines (Suiseng**^®^**, Lab. Hipra). PBS was used as control. Serum or fecal samples were diluted 1:100 or 1:20, respectively, in PBS. The results are shown as the mean levels for triplicates ± SD (* *p* < 0.05 for immunized mice vs. control; ^ *p* < 0.05 OMV-NP vs. free OMV).

Two-week-pregnant BALB/c mice were immunized orally with a single dose of OMV (0.2 mg/mouse) either free (OMV) or encapsulated into nanoparticles (OMV-NP). Control groups included mice immunized intraperitoneally with the commercial vaccine (Suiseng**^®^**) and non–immunized mice. The animals immunized with OMV-NP presented higher levels in serum of specific antibodies (IgG2a and IgG1) at week three post-immunization compared to sham-immunized mice (Figure 5). Specific fecal IgA levels were also higher in the OMV-NP group than in the commercial vaccine and significantly higher than those elicited by the non-encapsulated antigens (Figure 5).

**Figure 6:**
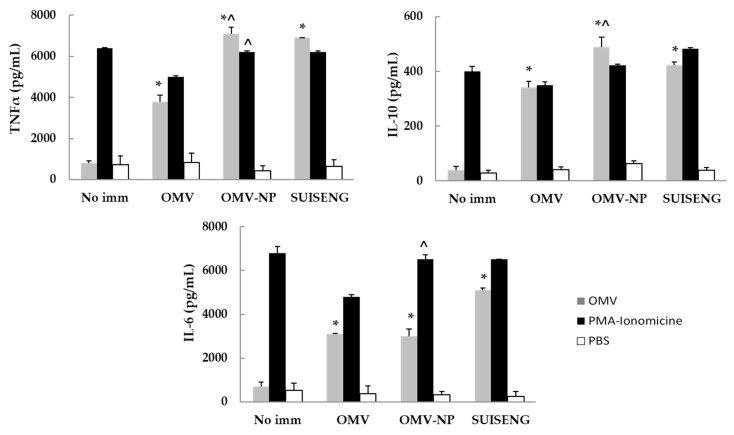
Splenic cytokine profile after immunization of pregnant BALB/c mice. Production of IL-6, IL-10 and TNFα by splenocytes restimulated with OMV in vitro was determined at day 28 post-immunization. The results are shown as the mean levels for triplicate determination ± SD (* *p* < 0.05 for immunized mice vs. control; ^ *p* < 0.05 OMV-NP vs. free OMV).

Production of IL-6, IL-10 and TNFα by splenocytes restimulated with OMV in vitro was determined at day 28 post-immunization (Figure 6). Results indicated that the encapsulation of OMV into zein nanoparticles induced an increase of IL-10 and TNFα cytokines, similar to the levels induced by the commercial vaccine, and significantly higher than the free OMV immunized group.

### 3.5. Antibody Response in the Offspring Pups and Protection against Challenge

**Figure 7:**
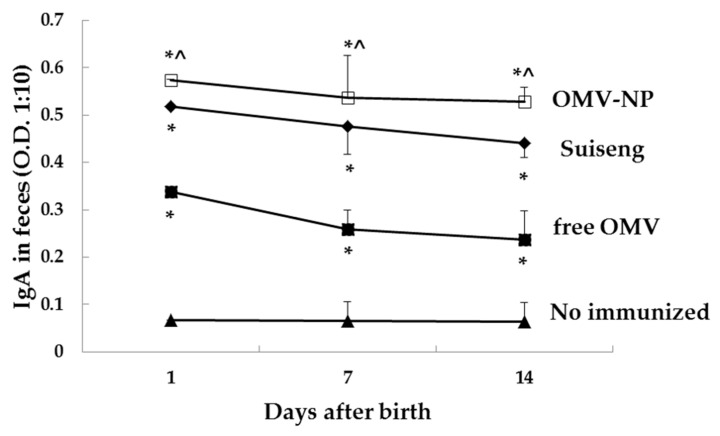
Antibody immune response in BALB/c mice pups from mothers immunized with OMV encapsulated into nanoparticles. Two-week-pregnant BALB/c mice were immunized orally with a single dose of OMV (0.2 mg) either free or encapsulated into nanoparticles (OMV-NP) or intraperitoneally with the commercial vaccines (Suiseng^®^, Lab. Hipra). At days 1, 7 and 14 after birth, IgA was determined in fecal samples (1:10 in PBS). The results are shown as the mean levels for triplicates ± SD (* *p* < 0.05 for immunized mice vs. control; ^ *p* < 0.05 OMV-NP vs. free OMV).

Fecal samples were taken from the pups at days 1, 7 and 14 after birth. Mice pups born from OMV-NP immunized mothers presented higher levels of fecal IgA than those by the commercial vaccine (Figure 7).

**Figure 8:**
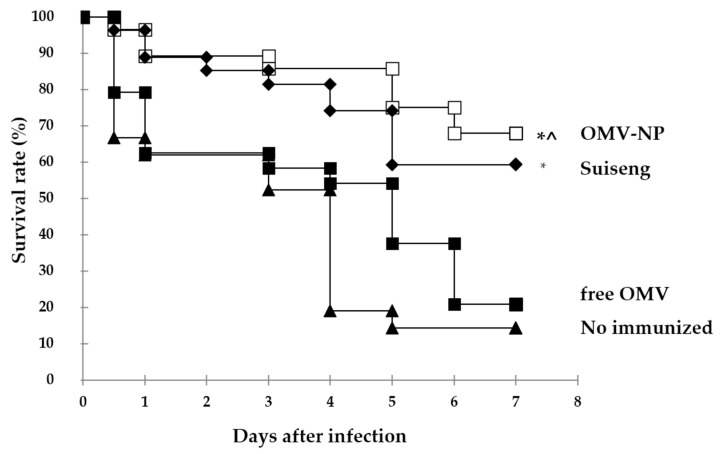
Effect of active immunization on the protection against F4-ETEC infection of BALB/c mice pups from mothers immunized with OMV encapsulated into nanoparticles. Two-week-pregnant BALB/c mice were immunized orally with a single dose of OMV (0.2 mg) either free or encapsulated into nanoparticles (OMV-NP) or intraperitoneally with the commercial vaccines (Suiseng^®^, Lab. Hipra). One week after birth, the pups were infected orally with a single dose of F4 *E. coli* ETEC (1.2 × 10^8^ CFU/mouse) resuspended in 100 µL PBS. Graphs indicate the percentage of mice that survived the infective challenge at the indicated days after immunization (*, *p* < 0.01, for immunized mice vs. control; ^ *p* < 0.05 OMV-NP vs. free OMV).

Finally, we evaluated the capacity of maternal vaccination to protect offspring against an experimental challenge of *Escherichia coli* F4 at day 7 after birth. The pups were studied along one week after the infection. Free OMV were not able to protect pups from infection (Figure 8). In contrast, OMV-NP and Suiseng**^®^** groups shown a protective capacity of 70% and 60%, respectively. In line with these results, pups from free OMV group presented clinical symptoms typical from intestinal infections such as diarrhea, weight loss and bristly hair. Mouse pups from OMV-NP and Suiseng**^®^** did not present any of these symptoms.

## 4. Discussion

In the present study, we show that pups from orally vaccinated mothers with OMV from the ETEC F4 serotype when encapsulated into nanoparticles were protected against challenge. Attenuated vaccines are contraindicated during pregnancy and OMV are becoming real vaccine candidates because of their safety and the immunogenic competence combining multiple virulence factors in a single particle [14]. The characterization studies of the OMV used in this work revealed the presence of relevant immunogenic components such as flagellin, OmpC/OmpA, lipoproteins and LPS, among others [15,16,17]. The existence of OMV on the surface of nanoparticles and the presence of YghJ, which degrades the major mucins MUC2 and MUC3 [18], could explain the capability of nanoparticles containing OMV to diffuse in the intestinal mucus as described previously [13].

OMV vaccine components can impact the course of infection by the activation of the innate immune response in the host. Thus, in this report we show a significant nitric oxide production by macrophages in response to in vitro stimulation with OMV, indicating the quality of the antigenic complex on relation with their potential protective immunogenic properties [19].

In addition, we evaluated the Th1/Th2 polarizing cytokine response after the stimulation of splenic cells of naïve BALB/c mice with OMV, showing its antigenic relevance for the development of the vaccine on the basis of the IFNγ (Th1) and IL-4 (Th2) levels after in vitro stimulation. These results are in agreement with those from Kim et al. using similar antigens [20]. The biological significance of IL-4 is related with the Th2 subset, IgA subclass and with the portal of entry of *Escherichia coli* ETEC. Our efforts are then directed to provide effective mucosal immunity to neutralize the access of the pathogen at the portal of entry. In this regard, the induction of significant specific IgA, the most abundant antibody isotype in the mucosae, may be of a great value [21,22].

However, these antigenic complexes are often scarcely immunogenic after oral immunization, probably due to the exposure to acid pH and the gastrointestinal enzymes and/or the inability to pass the mucus barrier. The use of nanoparticles offers protection to the loaded antigens against those harsh conditions of the gut [23]. Therefore, we used specific nanoparticles as oral adjuvants [24,25,26,27]**.** In fact, oral immunization requires the successful delivery of the intact and active antigen to the intestine avoiding degradation through the harsh environment in the stomach. Our previous literature indicated that GM-coated nanoparticles possess gastroresistant properties, adopting a compacted structure that minimizes the release of the loaded compound (i.e., OMV antigens) under acidic pH conditions. On the contrary, under neutral/basic pH conditions, zein nanoparticles swell facilitating the release of the cargo [28]. The nanoparticle formulation used in this work contained a core of zein and a corona of Gantrez-mannosamine in order to increase the adequate distribution and immunogenicity of the antigenic cargo. These characteristics have been observed for Gantrez^®^ AN nanoparticles when orally administered [29]. In addition, the use of Gantrez^®^ AN as the material to produce nanoparticles and/or polymer conjugates may be of interest to promote strong and balanced immune responses due to its capability as agonist for TLR2 and TLR4 [10]. The immunostimulant effect of Gantrez^®^ AN formulations was reported in vaccination studies against *Salmonella enterica* [30] or *Shigella flexneri* in mice [31], and vaccinated sows with this particular OMV-GM-Zein nanoparticle vaccine formulation showed specific IgG, IgA and IgM in serum [13]. In view of these results, we finally evaluated the capacity of this new vaccine to confer protection to offspring from vaccinated mothers.

Oral immunization of pregnant mice with nanoencapsulated OMV increases their specific antibodies levels in serum (IgG, IgG2a and IgA) as well as mucosal antibodies (IgA). The antibody response conferred by the nanoparticle adjuvant was also correlated with cellular immune response. Moreover, the born pups presented high specific levels of mucosal antibodies (IgA), which suggest that antibodies were efficiently transferred through lactation. In contrast, free OMV showed reduced ability to protect the offspring indicating the main role of the here used nanoparticles. These results support the adjuvanticity of these food-born nanoparticles in order to confer protection when encapsulating *E. coli* OMV. Finally, a major hurdle in oral immunization is that a higher dose of antigen is usually required to induce immunogenicity, but large doses also increase the risk of tolerance. This restriction can be circumvented using this sort of immunostimulating nanoparticles [10,11,32,33].

Vaccination of pregnant mothers has demonstrated to be a good approach to control endemic diseases [23]. The results on protection presented here together with those reported previously on immunogenicity in sows [13] support further studies of immunization with OMV into nanoparticles during pregnancy [34,35].

## 5. Conclusions

In this report ETEC OMV were tested alone or in formulation with GM-zein nanoparticles as a new vaccine candidate. Immunization of dam mice induced serum and mucosal antibody responses and elicited protection in offspring against an experimental challenge with the ETEC homologous strain. These results suggest that this nanoparticle formulation, which triggers mucosal immunity and results in passive immunity in offspring, is a good adjuvant candidate that merits further investigation.
